# Phase 2 multicenter study of pegaspargase in Japanese patients with previously untreated acute lymphoblastic leukemia

**DOI:** 10.1007/s12185-025-03976-4

**Published:** 2025-03-31

**Authors:** Katsuyoshi Koh, Yoshiyuki Kosaka, Yasuhiro Okamoto, Naoko Maeda, Atsushi Ogawa, Ryoji Kobayashi, Daisuke Hasegawa, Nobuhiro Koga, Adrien Tessier, Yelena Shvenke, Jian Zhu, Bouchra Benettaib, Keizo Horibe, Chitose Ogawa

**Affiliations:** 1 Department of Hematology/Oncology, Saitama Children’s Medical Center, 1-2, Shintoshin, Chuo-ku, Saitama-shi, Saitama 330-8777 Japan; 2 Division of Hematology and Oncology, Hyogo Prefectural Kobe Children’s Hospital, Hyogo, Japan; 3 Department of Pediatrics, Kagoshima University Hospital, Kagoshima, Japan; 4 Department of Pediatrics, NHO Nagoya Medical Center, Nagoya, Japan; 5 Department of Pediatrics, Niigata Cancer Center Hospital, Niigata, Japan; 6 Department of Hematology/Oncology for Children and Adolescents, Sapporo Hokuyu Hospital, Sapporo, Japan; 7 Department of Pediatrics, St. Luke’s International Hospital, Tokyo, Japan; 8 Nihon Servier, Tokyo, Japan; 9 Servier, Saclay, Paris, France; 10 Servier Pharmaceuticals, Boston, MA USA; 11 Clinical Research Center, NHO Nagoya Medical Center, Nagoya, Japan; 12 Department of Pediatric Oncology, National Cancer Center Hospital, Tokyo, Japan

**Keywords:** Acute lymphoblastic leukemia, Lyophilized pegaspargase, Pharmacokinetics, Safety, Efficacy

## Abstract

Pegaspargase is a pegylated formulation of *E. coli-*derived asparaginase, which when combined with multi-agent chemotherapy is an effective, well-established therapy for acute lymphoblastic leukemia (ALL). This study evaluated the efficacy, safety and pharmacokinetics of lyophilized pegaspargase in the Japanese population. The study had two parts; the primary endpoint for Part 1 was the incidence and nature of treatment-emergent adverse events (TEAEs), including those related to pegaspargase, to determine the number of patients who experience intolerable toxicity during a tolerability assessment period. The primary endpoint for Part 2 was the percentage of patients with plasma asparaginase activity of ≥ 0.1 IU/ml 14 days after administration of the first dose of lyophilized pegaspargase. All 26 patients included in the safety analysis experienced at least one TEAE. Frequently reported TEAEs related to lyophilized pegaspargase included decreases in blood fibrinogen, antithrombin III, white blood cell count, and platelet count. No deaths were reported. Plasma asparaginase activity reached ≥ 0.1 IU/ml 5 min after the first dose of lyophilized pegaspargase and was maintained for 14 days in all patients with evaluable samples. The results of this study show that lyophilized pegaspargase represents an effective and well-tolerated first-line treatment option in Japanese patients with ALL.

**Supplementary Information:**

The online version contains supplementary material available at 10.1007/s12185-025-03976-4.

## Introduction

Acute lymphoblastic leukemia (ALL) is the most common type of hematological malignancy in the pediatric population in Japan, comprising 46.6% of all childhood hematological malignancies and affecting ~ 400–500 children per year [1, 2]. Among children with ALL, the B-cell form accounts for ~ 85% of ALLs, while the remaining 15% are T-cell ALL [1, 3]. The treatment of ALL includes the long-term use of multi-agent chemotherapies, a cornerstone of which is L-asparaginase, which may be used in the induction, consolidation and maintenance phases of therapy [4, 5]. In Japan, patients with ALL are treated according to their risk group (standard, intermediate or high), and first-line therapy usually incorporates steroids, vincristine and asparaginase with or without anthracycline [6]. As standard L-asparaginase is a protein derived from *Escherichia coli *(*E. coli*), some patients experience allergic reactions such as anaphylactic shock, which prevents completion of L-asparaginase treatment [7].

Pegaspargase is a pegylated formulation of *E. coli-*derived asparaginase (i.e., L-asparaginase linked to polyethylene glycol [PEG]) that has a longer plasma half-life and lower immunogenicity than L-asparaginase [8]. The extension of the plasma half-life of pegaspargase allows a lower frequency of administration (every 14 days, compared with every other day with native L-asparaginase) [4]. Less frequent administration is associated with better quality of life, reduced pain and reduced anxiety [9].

Pegaspargase, when combined with multi-agent chemotherapy, is an effective and well-established therapy for ALL in adults [9–11] and in children [9, 12, 13]. Clinical development of pegaspargase was initially based on a liquid formulation. Subsequently, a lyophilized formulation was developed to improve the stability (shelf life of 36 and 8 months for the lyophilized and liquid formulations, respectively) and ensure continuous drug availability [14]. The lyophilized formulation of pegaspargase (Oncaspar^®^, Servier) has been approved in over 45 countries throughout the world, including a recent approval in Japan. This study was designed to evaluate the safety and pharmacokinetics (PK) of lyophilized pegaspargase in patients with ALL in the Japanese population.

Here we report PK, efficacy and safety results for the lyophilized formulation of pegaspargase in newly diagnosed, untreated children, adolescents and young adults with B-cell ALL in a Japanese population.

## Methods

### Study design

This was a two-part, single arm, multicenter, non-randomized, open-label, Phase 2 clinical study of lyophilized pegaspargase in Japanese patients with newly diagnosed, untreated ALL (NCT04067518). The study consisted of two parts: Part 1 recruited a small number of patients (n = 3) to assess tolerability and safety of a single dose of lyophilized pegaspargase, followed by repeated doses (the number of which was determined by the patient’s risk category) of lyophilized pegaspargase for up to 40 weeks. Upon review of the safety outcomes in Part 1 and determination that the dose of lyophilized pegaspargase was well tolerated, a larger number of patients entered Part 2 (n = 25) of the study to assess the efficacy, safety and PK profile of lyophilized pegaspargase for 45 weeks. The study was carried out between October 2019 and February 2022 at 10 centers in Japan.

This study was conducted in accordance with the ethical principles of the Declaration of Helsinki and Good Clinical Practice (GCP) guidelines; the protocol and the informed consent forms were approved by the Institutional Review Board (IRB) at each participating center. Written informed consent was provided by the patient or a legally acceptable representative before participation in Part 1 or 2 of the study.

### Patients

Patients with newly diagnosed, untreated precursor B-cell ALL (stratified into standard risk [SR], intermediate risk [IR] or high risk [HR] groups; Supplementary Table 1) who were aged 1 to ≤ 21 years at the time of informed consent with an Eastern Cooperative Oncology Group performance status (ECOG PS) 0 to 2 and no prior therapy for a malignant tumor such as chemotherapy/radiation therapy were eligible for study inclusion. Key exclusion criteria were: the presence of Down syndrome; mature B-cell ALL (e.g., Burkitt’s ALL); a currently active infection; poorly-controlled concurrent illness; pre-existing known coagulopathy (e.g., hemophilia and known protein S deficiency); and a history of pancreatitis. Additional eligibility criteria are described in Supplementary Table 2.

### Study treatments

**Fig. 1:**
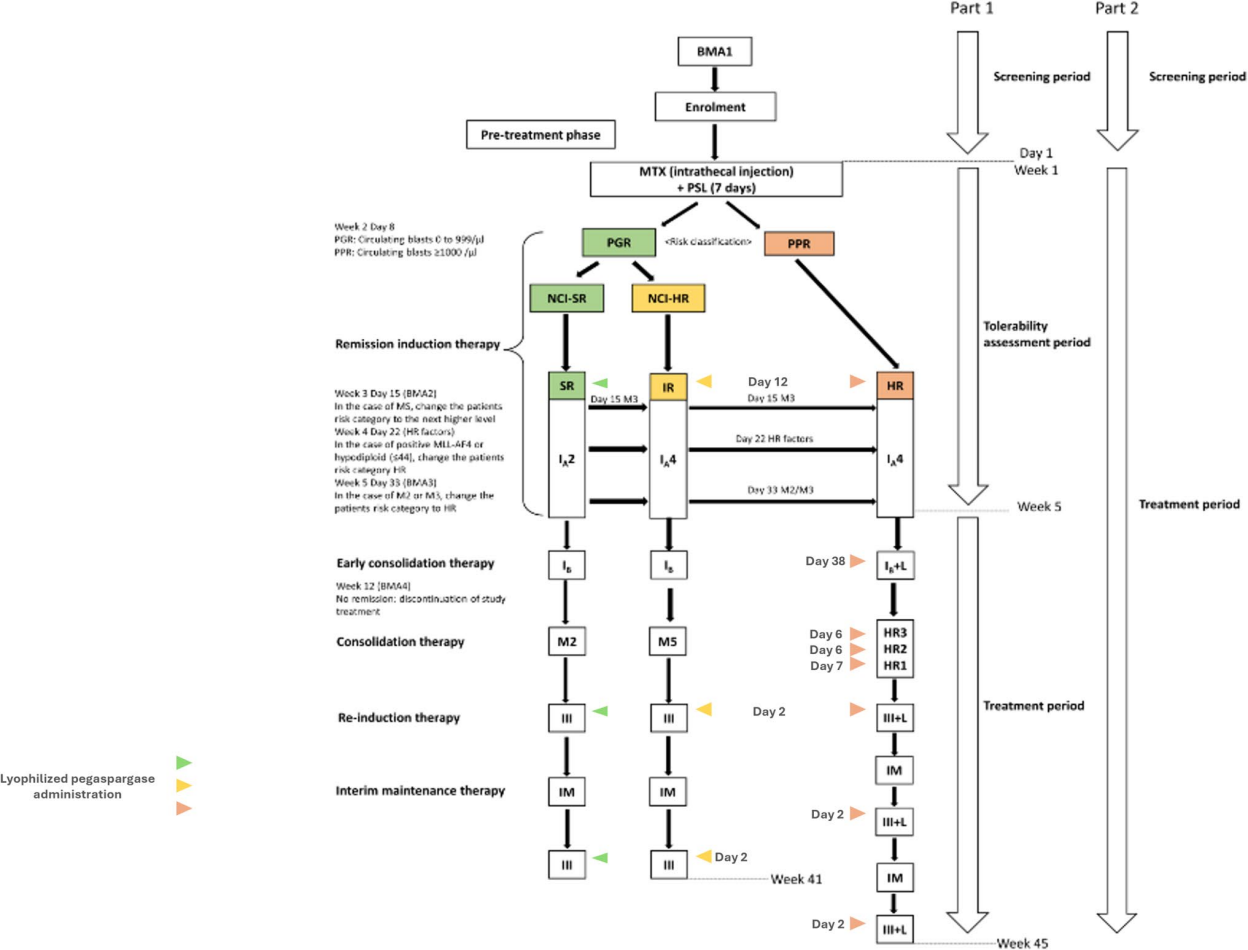
Treatment according to risk category and study design overview. *BMA*, bone marrow aspirations; *PGR*, prednisolone good responder; *PPR,* prednisolone poor responder; *I*_*A*_*2 and I*_*A*_*4*, remission induction therapy; *I*_*B*_* and I*_*B*_* + L*, early consolidation therapy; *M2, M5*, *HR1/2/3*, consolidation therapy; *IM*, interim maintenance therapy; *III, III + L*, re-induction therapy

Regimens for Part 1 and Part 2 of the study were based on the schedule used in Japan Children’s Cancer Group (JCCG) Study ALL-B12 (Fig. 1), with the tolerability assessment period in Part 1 consisting of a pre-treatment phase and remission induction therapy (Day 1–37) and the treatment period comprising early consolidation therapy, consolidation therapy, re-induction therapy, and interim maintenance therapy [2]. Part 1 planned to enroll 3–6 patients. Patients in Part 1 initially received a single dose of lyophilized pegaspargase on Day 12 and were assessed for tolerability until Day 37. If 1 of the first 3 patients experienced intolerable toxicity (defined as pegaspargase-related persistent grade ≥ 3 adverse events [AEs], any AE considered intolerable by the treating physician, or any AE that led to interruption of therapy for ≥ 28 days), an additional 3 patients were enrolled. If ≥ 2 of the first 3 patients or ≥ 2 of the 6 patients experienced intolerable toxicity the possibility of dose reduction was discussed with the trial funder before any other patients were enrolled. If there was no intolerable toxicity during Days 1–37 of Part 1, patients received repeated doses of lyophilized pegaspargase in the treatment period of Part 1 and Part 2 used the same dose as Part 1. Patients categorized as SR or IR received 2 further doses (on Day 2 of each re-induction therapy; a total of 3 doses; Fig. 1) of lyophilized pegaspargase for 41 weeks. Patients categorized as HR received 7 further doses (on Day 38, Day 6 or 7 of each consolidation therapy, and Day 2 of each re-induction therapy; a total of 8 doses; Fig. 1) of lyophilized pegaspargase for 45 weeks (Fig. 1). Part 2 followed the same regimen as Part 1.

The intravenous (IV) dose of lyophilized pegaspargase was 2500 IU/m^2^ for individuals with a body surface area (BSA) ≥ 0.6 m^2^, or 82.5 IU/kg for those with a BSA < 0.6 m^2^. BSA was calculated based on the height and weight measured on the start day of each treatment phase (including Day 8 of remission induction therapy and Day 36 of early consolidation therapy). The drugs for combination chemotherapy other than lyophilized pegaspargase were administered according to the regimens and doses used in JCCG Study ALL-B12 [2]. The backbone therapy drugs used in this study are listed in Supplementary Table 3.

### Assessments

In both Parts of the study, the assessment schedule for all patients was the same from enrolment to Week 5. At screening and on Days 1, 8, 12, 15, 22, and 29 all patients were assessed for vital signs, ECOG PS, and had hematological and chemistry assessments, immunoserological tests, and urinalysis.

From Week 6 to treatment completion the schedule was different for patients categorized as SR/IR and patients categorized as HR. In the SR/IR group, assessments for vital signs, ECOG PS, hematology and chemistry were once weekly from Weeks 6–36 and Week 38 to study end, and twice weekly in Week 37. Immunoserological tests and urinalysis were conducted on Weeks 6, 12, 22, 27, and 37 (urinalysis twice in Weeks 27 and 37). In the HR group, assessments for vital signs, ECOG PS, hematology and chemistry were once weekly from Weeks 6 to 14, 16 to 17, 19 to 20, 22 to 30, 32 to 40, and 42 to study end, and twice weekly in Weeks 15, 18, 21, 31, and 41. Immunoserological tests and urinalysis were conducted on Weeks 6, 12, 21, 26, 31, 36 and 41 (urinalysis twice in Weeks 6, 12, 21, 31, 41, and once in Weeks 15, 18, 26, and 36) (Fig. 1).

At each visit from screening to end of study, patients were assessed for AEs and had a review of concomitant medications and therapies.

Plasma samples for the measurement of plasma asparaginase activity that were used for PK analysis were collected during the remission induction therapy before dosing, then 5 min, 4, 24 and 48 h, and 4, 11, 14, 18 and 25 days after pegaspargase dosing. During the re-induction therapy, PK samples were collected before dosing, then 11, 14 and 25 days after pegaspargase dosing. A PK sample was collected at treatment completion. Immunogenicity assessment included the detection of anti-drug antibodies (ADA; i.e., anti-pegaspargase antibodies) and antibodies against PEG in samples taken before and 25 days after pegaspargase dosing during the remission induction therapy, before pegaspargase dosing during the re-induction therapy and at treatment completion (Fig. 1).

### Study endpoints

The primary endpoint for Part 1 was the incidence and nature of treatment-emergent AEs (TEAEs), including lyophilized pegaspargase related TEAEs, in order to determine the number of patients who experience intolerable toxicity during a tolerability assessment period (the 25 days after the first dose of lyophilized pegaspargase). Intolerable toxicity was defined as the following: grade 3 or higher toxicity (that did not improve to grade 2 or lower by day 37), with the exception of adverse drug reactions related to other treatment; any expected toxicity that was more severe than usual and judged by the physician to be intolerable; and any pegaspargase-related TEAE that led to interruption of early consolidation therapy for ≥ 28 days.

The primary endpoint for Part 2 was the percentage of patients with plasma asparaginase activity of ≥ 0.1 IU/ml 14 days (336 h) after administration of the first dose of lyophilized pegaspargase. Secondary endpoints for Part 1 included the percentage of patients with plasma asparaginase activity of ≥ 0.1 IU/ml 14 days (336 h) after administration of the first dose of lyophilized pegaspargase. Additional secondary endpoints in both Part 1 and 2 were: safety (incidence and nature of TEAEs and lyophilized pegaspargase-related TEAEs, laboratory values, and vital signs), PK parameters, evaluation of ADA and anti-PEG antibodies, and overall survival (OS) and event-free survival (EFS) rates at 1 year after lyophilized pegaspargase treatment initiation. Exploratory endpoints encompassed rates of complete remission (CR) and complete remission with incomplete blood count recovery (CRi) and overall response rate (ORR; CR + CRi) at the end of remission induction therapy and early consolidation therapy.

### Statistical analysis

Target enrollment for Part 2 was 22 patients, which was based, from previous studies of lyophilized pegaspargase [15], on an expected 90.9% of patients achieving a plasma asparaginase activity ≥ 0.1 IU/ml 14 days (336 h) after the first dose of lyophilized pegaspargase with a 95% confidence interval (CI) of 70.8–98.9%.

As this is the first study of lyophilized pegaspargase in Japanese patients, all analyses were performed separately for Part 1 and Part 2 according to the approved protocol. Safety analyses were performed in the safety analysis set, which comprised all patients who received ≥ 1 dose of lyophilized pegaspargase in Part 1 or Part 2. Descriptive analyses on efficacy endpoints in Part 1 were based on the safety analysis set. All efficacy analyses for Part 2 were undertaken in the full analysis set (defined as all enrolled patients who received lyophilized pegaspargase in Part 2). Other evaluations were conducted in the PK analysis set (all patients enrolled in Part 1 or Part 2 who had received first dose of lyophilized pegaspargase at remission induction therapy and who were evaluable for non-compartmental assessment) and immunogenicity analysis set (all patients who received ≥ 1 dose of lyophilized pegaspargase in Part 1 or Part 2 and had ≥ 1 evaluable post-dose sample).

The number and percentage of patients with a plasma asparaginase activity of ≥ 0.1 IU/ml 14 days (336 h) after the first dose of lyophilized pegaspargase was assessed in Part 1 and Part 2, with the corresponding 95% CIs calculated based on the Clopper-Pearson method. Plasma or serum asparaginase activity is an effective surrogate endpoint for the clinical efficacy of lyophilized pegaspargase, and a plasma/serum asparaginase activity of ≥ 0.1 IU/ml is the threshold for securing sustained asparagine depletion [16]. Patients with partial asparaginase activity data, protocol deviations, or events with the potential to affect PK were evaluated on a case-by-case basis to determine if sufficient data were available for reliable summarization of asparaginase activity and estimation of PK parameters, which were calculated using non-compartmental methods.

OS and EFS at one year after the first dose of lyophilized pegaspargase were calculated based on the Kaplan–Meier method, and the corresponding 95% CIs were also assessed. The CR rate, CRi rate, and ORR at the end of remission induction therapy and early consolidation therapy, and the corresponding 95% CIs were also assessed.

Frequencies of TEAEs, lyophilized pegaspargase-related TEAEs, and TEAEs of special interest (‘hypersensitivity’, ‘acute pancreatitis’, and ‘embolic and thrombotic events’) were summarized based on the Medical Dictionary for Regulatory Activities (MedDRA, version 24.1). Changes in laboratory parameters, vital signs, and echocardiography parameters at each time point are presented descriptively. Persistence of ADA or anti-PEG antibodies in the immunogenicity analysis set was evaluated based on consecutive results after treatment. The number and percentage of patients with seroconversions by visit upon treatment are presented.

### Population pharmacokinetics analysis

A population pharmacokinetic (PPK) analysis was performed on the pegaspargase activity data from this study (n = 26, all considered Japanese patients), pooled with data from three previously published studies (n = 225, all considered non-Japanese patients) where a liquid formulation of pegaspargase 2500 IU/m^2^ was administered through IV infusion [15, 17, 18] or intramuscular (IM) injection [13].

A PPK model was developed using the data from the four studies. The PPK model was a one-compartment model with first-order absorption and two bioavailability estimates for the first and later IM doses, and a parallel saturable and linear elimination. The PPK model used NONMEM version 7.5 and the first-order conditional estimation method with interaction (FOCEI) for parameter estimation. Potential covariate-parameter relationships were evaluated using the stepwise covariate model building procedure with adaptive scope reduction [19]. The model included the impact of BSA on the central volume of distribution (V_c_), the linear elimination clearance (CL) and the maximum elimination capacity of the saturable elimination (V_max_). Sex was tested on V_c_, CL and V_max_, the presence of ADA was tested on CL and V_max_, and the population (Japanese and non-Japanese) was tested on V_c_ and CL. The model was evaluated using the relative standard errors of parameter estimates, and graphical diagnostics including goodness of fit plots and visual predictive checks.

From the PPK model, the impact of covariates on PK exposure parameters (C_max_ and area under the asparaginase activity-time curve up to 25 days [AUC_0-25d_] after a single dose of pegaspargase 2500 IU/m^2^) was evaluated through forest plots. The precision in the covariate effects was based on model parameters sampled 250 times in the variance–covariance matrix obtained from NONMEM.

## Results

### Patients

Of 28 patients screened (3 in Part 1 and 25 in Part 2), two patients failed screening or did not meet inclusion criteria, and therefore, 26 patients (3 in Part 1 and 23 in Part 2) received at least one dose of lyophilized pegaspargase with the combination of backbone therapy drugs (safety analysis set). Twenty-three patients were included in the full analysis set (Part 2: 23 patients), 25 patients were included in the immunogenicity analysis set (Part 1: three patients, Part 2: 22 patients), and 26 patients were included in the PK analysis set (Part 1: three patients, Part 2: 23 patients). Patients had a median age of 4.8 years and 73.1% were under 10 years of age (Table 1). There were 25 patients in the SR/IR group and one patient in the HR group. In the SR/IR group, 22/25 patients received 3 doses of lyophilized pegaspargase as scheduled in the protocol. Two patients received 1 dose and one patient received 2 doses due to treatment discontinuation. Patients in the SR/IR group received a mean (standard deviation [SD]) cumulative lyophilized pegaspargase dose of 6154.6 (2911.9) IU over 267 (86.7) days. Overall, the dose of lyophilized pegaspargase was administered as planned. For the one patient in the HR group, only 3 doses were administered due to treatment discontinuation. The patient in the HR group received a mean cumulative lyophilized pegaspargase dose of 11,300 IU over 95 days. Three of the four patients discontinuing treatment, discontinued lyophilized pegaspargase due to AEs related to treatment (one patient in Part 1 had grade 3 non-serious anaphylactic reaction, one patient in Part 2 had grade 3 non-serious pancreatitis and one patient in Part 2 had grade 4 serious pancreatitis acute). The other patient discontinuing treatment in Part 2 had grade 3 serious posterior reversible encephalopathy syndrome, not related to pegaspargase, that led to discontinuation of backbone therapy drug. These discontinuations are presented in more detail below.

**Table 1:** Baseline characteristics

Characteristic	Patients (n = 26)
Age at informed consent, years, median (range)	4.8 (1.0–17.0)
Sex, n (%)	
Male	13 (50.0)
Weight, kg	
Median (range)	15.8 (11.2–65.2)
Height, cm	
Median (range)	107.5 (78.0–172.0)
BSA, m^2^, median (range)	0.68 (0.49–1.75)
n (%)	
≥ 0.60	21 (80.8)
< 0.60	5 (19.2)
WBC count, × 10^9^/l, median (range)	3.50 (0.7–83.9)
n (%)	
< 50	25 (96.2)
≥ 50	1 (3.8)
*NUDT15* polymorphism	
Arg/Arg	22 (84.6)
Cys/Cys	1 (3.8)
Other homozygous or heterozygous genotype	3 (11.5)

*BSA*, body surface area; *NUDT15*, nudix hydrolase 15; *WBC*, white blood cell

### Primary endpoint of Part 1 and determination of dose for Part 2

All three patients in Part 1 experienced at least 1 TEAE related to pegaspargase. No patients experienced an intolerable toxicity during the tolerability assessment period in Part 1 (primary endpoint for Part 1), and therefore, the IV dose of lyophilized pegaspargase used in Part 2 was the same as the dose in Part 1. All three patients experienced blood fibrinogen decrease, antithrombin III decrease, plasmin inhibitor decrease, plasminogen decrease, and hyperlipidemia. One patient experienced a non-serious TEAE considered related to lyophilized pegaspargase (grade 3 anaphylactic reaction at second administration of pegaspargase 167 days after starting treatment) leading to discontinuation of lyophilized pegaspargase.

### Efficacy

In Part 1 all three patients (100%; 95% CI 29.2%, 100.0%) achieved a plasma asparaginase activity of ≥ 0.1 IU/ml 14 days after the first dose of lyophilized pegaspargase. For the second and third dose of lyophilized pegaspargase, all evaluable patients achieved a plasma asparaginase activity of ≥ 0.1 IU/ml 14 days after administration (100% for both second and third doses). In Part 2, all 23 patients (100%; 95% CI 85.2%, 100.0%) in the full analysis set achieved a plasma asparaginase activity of ≥ 0.1 IU/ml 14 days after the first dose of lyophilized pegaspargase. Plasma asparaginase activity reached ≥ 0.1 IU/ml at 5 min after the first dose of lyophilized pegaspargase and was maintained for 14 days in all patients (100.0%) with evaluable samples. One of 22 patients (4.5%) had a decreased plasma asparaginase activity of < 0.1 IU/ml 18 days after the first dose of lyophilized pegaspargase in Part 2 (data during re-induction therapy assessed after receiving COVID-19 vaccine was excluded from analysis). For the second and third dose of lyophilized pegaspargase, the majority of evaluable patients achieved a plasma asparaginase activity of ≥ 0.1 IU/ml 14 days after administration (85.0% for the second dose and 94.4% for the third dose).

In Part 1 and 2, OS and EFS rates at one year after the first dose of lyophilized pegaspargase were 100%. ORR for both Part 1 and Part 2 was also 100% in analyzed patients based on CR and CRi rates (Supplementary Table 4).

### Pharmacokinetics

**Fig. 2:**
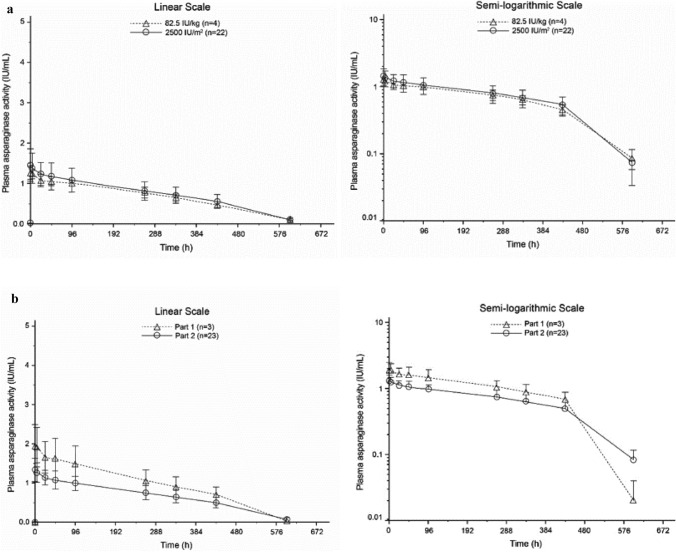
Mean ( SD) plasma asparaginase activity-time profiles on linear and semi-logarithmic scales **a** by dose and **b** by Part—remission induction (PK analysis set). *h*, hours; *PK*, pharmacokinetic; *SD*, standard deviation

The PK analysis included 26 patients (three patients in Part 1; 23 patients in Part 2). PK parameters by BSA thresholds for lyophilized pegaspargase dosing in Part 1 and Part 2 of the study are summarized in Table 2. In patients positive for ADA or anti-PEG antibody before the start of lyophilized pegaspargase administration (see below), C_max_, t_max_, AUC_0-t_, t_1/2_ and other PK variables were similar to those measured in the rest of the population. In general, the mean plasma asparaginase activity-time profiles were similar in the two Parts of the study, by BSA thresholds for lyophilized pegaspargase dosing (Supplementary Table 5; Fig. 2), and by ADA or anti-PEG antibody status at baseline. Higher variability was observed for T_1/2_ in the 2500 IU/m^2^ dose versus the 82.5 IU/kg dose (82.7% coefficient of variation [CV] vs 24.8% CV).

**Table 2:** Summary of PK parameters for lyophilized pegaspargase by BSA dosing thresholds

PK parameter	BSA dosing threshold	
	2500 IU/m^2^ (BSA ≥ 0.6 m^2^) (n = 22)	82.5 IU/kg (BSA < 0.6 m^2^) (n = 4)
C_max_, IU/ml, arithmetic mean (SD)	1.456 (0.384)	1.256 (0.146)
t_max_, h, median (range)	1.74 (1.42–48.6)	1.78 (1.63–5.0)
AUC_0__-__t_, h*IU/ml, arithmetic mean (SD)	402.0 (92.837)	375.2 (51.628)
AUC_0__-__inf_, h*IU/ml, arithmetic mean (SD)	405.4 (85.753)	387.3 (55.135)
t_1/2_, h, arithmetic mean (SD)	121.0 (100.16)	92.52 (22.964)
CL, L/h, arithmetic mean (SD)	0.006695 (0.0034763)	0.002463 (0.00039535)
V_ss_, L, arithmetic mean (SD)	1.520 (0.84159)	0.5584 (0.059550)

*AUC*_*0-t*_, area under the curve from time 0 to t; *AUC*_*0-inf*_, area under the curve from time 0 to infinity; *BSA*, body surface area; *C*_*max*_, maximum observed plasma asparaginase activity; *CL*, clearance; *SD*, standard deviation; *t*_*max*_, time to peak observed plasma asparaginase activity; *t*_*1/2*_, elimination half-life of plasma asparaginase activity; *V*_*ss*_, volume of distribution at steady state

### Safety and immunogenicity

All 26 patients included in the safety analysis experienced at least one TEAE, TEAE related to lyophilized pegaspargase, or a TEAE of grade ≥ 3 (Table 3; Supplementary Table 6). The most frequently reported TEAEs were consistent between the < 10-years-old group and the ≥ 10-years-old group. Overall, 11 (42.3%) patients across both Parts of the study had at least one serious TEAE, of which, grade 1 vomiting and grade 4 acute pancreatitis were considered related to lyophilized pegaspargase. The serious TEAE of vomiting occurred 310 days after starting treatment with lyophilized pegaspargase and resolved 5 days after onset without treatment and with no dose adjustments of lyophilized pegaspargase. The serious TEAE of acute pancreatitis occurred 125 days after starting treatment with lyophilized pegaspargase on Day 2 of the HR2 phase of consolidation therapy, study drug was discontinued immediately, and the event resolved 24 days after onset. TEAEs of special interest were hypersensitivity, acute pancreatitis, and embolic and thrombotic events. One patient (3.8%) in Part 1 experienced grade 3 non-serious anaphylactic reaction that resulted in permanent discontinuation of the study drug (Table 3). This non-serious anaphylactic reaction occurred 167 days after starting treatment with lyophilized pegaspargase, study drug was discontinued immediately and permanently, and the event resolved on the same day. In Part 2, one patient (3.8%) experienced grade 3 non-serious pancreatitis 26 days after starting treatment with lyophilized pegaspargase, study drug was discontinued immediately, and the patient discontinued from the study, but the treating physician considered the pancreatitis to be resolving at time of discontinuation. One patient (3.8%) experienced grade 4 serious acute pancreatitis (as described above) (Table 3). Both these cases of pancreatitis resulted in permanent discontinuation of the study drug at the onset of the AE (Table 3). One patient (3.8%) experienced a TEAE of grade 3 serious posterior reversible encephalopathy syndrome, not related to lyophilized pegaspargase that led to discontinuation of backbone treatment (Table 3). In addition, one patient (3.8%) in Part 2 had a TEAE of disseminated intravascular coagulation not related to the study drug. One patient had an accidental overdose of lyophilized pegaspargase, the patient received 2800 IU on day 12 of the induction cycle, the event was grade 1 and did not require any intervention. No deaths were reported.

**Table 3:** Treatment emergent adverse events

	Part 1 (n = 3) n (%)	Part 2 (n = 23) n (%)	Total (n = 26) n (%)
Patients with at least 1 TEAE	3 (100.0)	23 (100.0)	26 (100.0)
Grade 3 + TEAEs	3 (100.0)	23 (100.0)	26 (100.0)
TEAEs related to lyophilized pegaspargase	3 (100.0)	23 (100.0)	26 (100.0)
Serious TEAEs	1 (33.3)	10 (43.5)	11 (42.3)
TEAEs leading to death	0 (0.0)	0 (0.0)	0 (0.0)
TEAEs leading to discontinuation of lyophilized pegaspargase			
Pancreatitis grade 3	0 (0.0)	1 (4.3)	1 (3.8)
Pancreatitis acute grade 4	0 (0.0)	1 (4.3)	1 (3.8)
Anaphylactic reaction grade 3	1 (33.3)	0 (0.0)	1 (3.8)
TEAEs leading to discontinuation of backbone therapy drug (other than lyophilized pegaspargase)			
Posterior reversible encephalopathy syndrome	0 (0)	1 (4.3)	1 (3.8)
TEAEs reported in > 70% of patients			
Anemia	3 (100.0)	21 (91.3)	24 (92.3)
Febrile neutropenia	2 (66.7)	18 (78.3)	20 (76.9)
Vomiting	2 (66.7)	19 (82.6)	21 (80.8)
Constipation	2 (66.7)	17 (73.9)	19 (73.1)
Platelet count decreased	3 (100.0)	22 (95.7)	25 (96.2)
White blood cell count decreased	3 (100.0)	21 (91.3)	24 (92.3)
Blood fibrinogen decreased	3 (100.0)	16 (69.6)	19 (73.1)

Adverse events are coded using the MedDRA dictionary, version 24.1. Patients with multiple events within a category are counted only once for that category. *TEAE*, treatment-emergent adverse event

There were no clinically meaningful changes in laboratory variables (blood chemistry, hematology, coagulation, and urinalysis), and there were no clinically meaningful findings in vital signs, ECG interpretations, or any other measurement.

In the immunogenicity analysis population, four patients (16.0%) were positive for ADA including two patients (8.0%) who were also positive for anti-PEG antibody before the start of lyophilized pegaspargase administration. Seroconversion for ADAs was identified in two (8.0%) patients, both were positive for ADA and anti-PEG (one patient at study Day 315, and the other at study Day 298). There was no loss of asparaginase activity after the seroconversion in these two patients.

### Population pharmacokinetics analysis

**Fig. 3:**
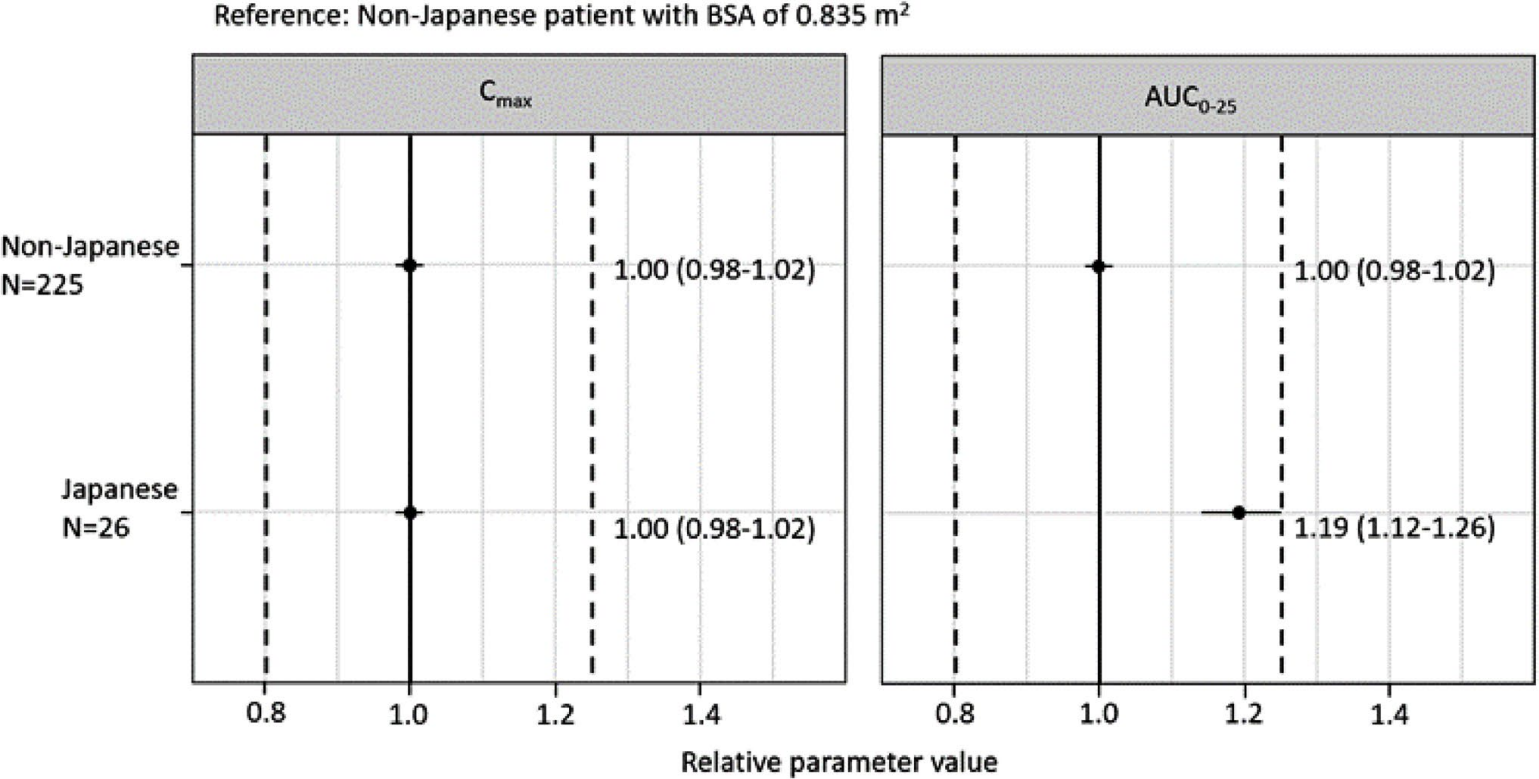
Forest plots illustrating the effect of the covariate Japanese versus non-Japanese population on asparaginase activity parameters C_max_ and AUC_0−25d_ after a single dose of pegaspargase 2500 IU/m^2^. Dots and error bars represent the median of the predicted relative change from the reference patient and its associated 90% CI. The parameter values for a reference non-Japanese patient with BSA of 0.835 m^2^ are shown by the solid vertical lines; the dashed vertical lines indicate the 80–125% margins relative to the reference patient. *AUC*_*0−25d*_, area under the asparaginase activity-time curve up to 25 days post-dose; *BSA,* body surface area; *CI,* confidence interval

A difference between the Japanese (n = 26) and the non-Japanese population (n = 225) was identified on CL (41% lower in the Japanese population) and the asparaginase concentration with activity at 50% of the V_max_ (K_m_; 83% lower in the Japanese population). Forest plots (Fig. 3) illustrated that, after a single pegaspargase 2500 IU/m^2^ dose, C_max_ was similar in both Japanese and non-Japanese population, while the Japanese population may achieve higher AUC_0-25d_. However, this impact on AUC_0-25d_ was considered not clinically meaningful as the median change was included in the 80–125% interval.

## Discussion

This single arm, Phase 2 study is the first clinical trial of pegaspargase in Japan and the first clinical trial specifically focused on lyophilized pegaspargase. In this study of lyophilized pegaspargase in Japanese patients with newly diagnosed, untreated ALL, all 26 patients achieved a plasma asparaginase activity of ≥ 0.1 IU/ml, the threshold for securing sustained asparagine depletion [16], rapidly (within 5 min), which was maintained until 14 days after the first dose of lyophilized pegaspargase.

Consistent with the maintenance of asparagine depletion and classification of SR/IR in all but one patient in the study, both survival rate and EFS rate at 1 year after the first dose of lyophilized pegaspargase was 100% across Part 1 and 2 of the study. PK data from this study demonstrated that exposure to lyophilized pegaspargase, peak plasma levels, time to peak plasma levels and elimination half-life were generally similar for both doses administered (82.5 IU/kg or 2500 IU/m^2^). The elimination half-life of lyophilized pegaspargase was approximately 4 to 5 days. Mean CL and V_ss_ were approximately 2.7-fold higher following an IV infusion of 2500 IU/m^2^ for patients with a BSA ≥ 0.6 m^2^ when compared with an IV infusion of 82.5 IU/kg for patients with a BSA < 0.6 m^2^. The higher CL and V_ss_ values were expected for patients with larger body sizes (i.e., BSA ≥ 0.6 m^2^), given the approximate directly proportional relationship between BSA and PK parameters such as clearance and distribution volume, as shown in previous population PK analyses [20, 21]. These results support the use of BSA- or body weight-based dose for lyophilized pegaspargase in order to limit the variability in exposure to pegaspargase.

The PPK analysis performed by pooling asparaginase activity data from this Phase 2 study in Japan and previous non-Japanese studies [13, 15, 17, 18] confirmed the relationship between BSA and PK parameters. The higher exposure (AUC_0−25d_) identified in the Japanese population was not considered clinically meaningful. It should be noted that the Phase 2 study in Japan used the lyophilized formulation of pegaspargase, while the previously published studies used the liquid formulation. Consequently, we cannot differentiate between the lyophilized formulation and Japanese population as reasons for the different exposure, and these results should be interpreted carefully.

The primary safety endpoint of this study was tolerability (Part 1), and no patients in Part 1 experienced an intolerable toxicity, and so the same dose of lyophilized pegaspargase was used in Part 2. All patients experienced at least one TEAE related to lyophilized pegaspargase, with the most frequent being decreases in blood fibrinogen, antithrombin III, white blood cell count and platelet count, all of which are consistent with the known safety profile of pegaspargase. The rates and nature of TEAEs of special interest (hypersensitivity, acute pancreatitis and embolic and thrombotic events) were consistent with the known safety profile of asparaginase [22]. No new safety concerns were identified with lyophilized pegaspargase, and no deaths were reported. There was no loss of asparaginase activity that could be related to the development of ADA in the study. Furthermore, lyophilized pegaspargase is used with several concomitant medications, so it is important to consider the fact that TEAEs could be related to not only lyophilized pegaspargase, but also other medications used in combination. Blood and lymphatic system disorders and skin and subcutaneous tissue disorders could be related to anthracycline, endocrine or psychiatric disorders could be related to steroids, hepatobiliary, musculoskeletal or connective tissue disorders could be related to vincristine, and nervous system disorders could be related to methotrexate.

In Japan, L-asparaginase is approved for use in pediatric ALL, but is associated with allergic reactions in some patients, meaning they need to discontinue this element of the therapy, which impacts on their prognosis. Two patients in the current study were positive for anti-PEG antibody before the start of lyophilized pegaspargase which could be due to previous exposure to pegylated products.

As the half-life of L-asparaginase is short (~ 1.2 days), it needs to be administered every 2–3 days, whereas lyophilized pegaspargase can be administered every two weeks, and therefore, lyophilized pegaspargase could reduce the burden of treatment for patients, as well as reducing resources and costs for healthcare professionals, as a considerably lower number of doses are needed during remission induction therapy and consolidation therapy for lyophilized pegaspargase. In fact, a 2019 UK cost-effectiveness study of patients with newly diagnosed ALL demonstrated that the costs of first-line lyophilized pegaspargase were lower compared with L-asparaginase and were associated with more quality-adjusted life year gains [23]. Similar findings have been demonstrated in Greece [24], and there may be potential costs savings in Japan, but this would need to be verified in future studies or pharmaco-economic models. Lyophilized pegaspargase can therefore improve the safety of treatment, along with an improvement in quality of life due to a much lower frequency of administration, and this is likely to be more pertinent in children, who may find every other day injections distressing and painful. Lyophilized pegaspargase may be a welcome addition to the available first-line treatment options for childhood precursor B-cell ALL in the Japanese population.

In this study, one patient (3.8%) was included in the HR group, which is slightly lower, but not dissimilar to, the frequency of 11.3% reported among pediatric patients (200 of 1766) assigned to the HR group in the Japanese ALL-B12 study [25]. The reasons for this are unclear, but this may be an artefact of the smaller sample size used in this study. Although the sample size was estimated based on previous study findings, these results in an overall population of 26 patients warrant further investigation in larger studies. Another potential limitation of this study was that certain therapeutic effects (e.g., minimal residual disease) were not investigated.

## Conclusions

Consistent with previous studies, lyophilized pegaspargase demonstrated efficacy, with no new safety signals, and the PK of both doses of pegaspargase in the current study were generally similar. This suggests that lyophilized pegaspargase potentially represents an efficacious and well tolerated first-line treatment option in Japanese patients with pediatric precursor B-cell ALL.

## Supplementary Information

Below is the link to the electronic supplementary material.

Supplementary file1 (DOCX 44 KB)

## Data Availability

Data are available on reasonable request. All data relevant to the study are included in the article or uploaded as supplementary information. De-identified patient- and/or study-level clinical trial data, including the clinical study report and study protocol, will be shared, in line with the Servier Data-Sharing Policy (available at https://clinicaltrials.servier.com/data-request-portal/).
