# Supplementary material for: Phase 2 multicenter study of pegaspargase in Japanese patients with previously untreated acute lymphoblastic leukemia
Source: Int J Hematol. 2025 Mar 31;122(2):284–94. doi: 10.1007/s12185-025-03976-4 (PMC12304028; doi:10.1007/s12185-025-03976-4)
Supplement: Supplementary file 1 — Supplementary file1 (DOCX 44 KB) [file 12185_2025_3976_MOESM1_ESM.docx]

**Phase 2 multicenter study of pegaspargase in Japanese patients with previously untreated acute lymphoblastic leukemia**

**SUPPLEMENTARY INFORMATION**

**Supplementary Table 1**. Criteria for risk classification*

| **Standard** | Patient meets **all** the following criteria:   - Not categorized as HR according to this classification - Prednisolone-good responder (PGR) - Categorized as SR per NCI classification (NCI-SR) - M1 or M2 marrow^a^ at bone marrow examination on Day 15 of remission induction therapy (BMA2) |
| --- | --- |
| **Intermediate** | Patient meets **any** of the following criteria:   - Categorized as other than HR according to this classification, PGR, and HR per NCI classification (NCI-HR) - Categorized as PGR and NCI-SR; and M3 marrow^a^ at bone marrow examination on Day 15 of remission induction therapy (BMA2) |
| **High** | Patient meets **one or more** of the following criteria:   - CNS-3 on Day 1 of the pre-treatment phase - Categorized as a prednisolone-poor responder (PPR) on Day 8 of remission induction therapy - Categorized as PGR and NCI-HR; and M3 marrow^a^ on Day 15 of remission induction therapy (BMA2) - Assessed as having refractory disease at BMA3 - KMT2A-AFF1 (MLL-AF4) fusion gene-positive (to be determined before the start of treatment on Day 22 of remission induction therapy) - Hypodiploid (≤44 chromosomes) (to be determined before the start of treatment on Day 22 of remission induction therapy) |

*Risk groups were stratified according to a comprehensive assessment from the pre-treatment phase through to the remission induction therapy phase.

a: Bone marrow status is categorized as follows according to the percentage of blasts in bone marrow: M1 marrow: <5%; M2 marrow: 5% to ≤25%; M3 marrow: >25%

**Supplementary Table 2.** Additional eligibility criteria

| **Inclusion criteria**   - AST and ALT ≤10 × age-specific upper limit of normal - Direct bilirubin <1.5 mg/dl - Serum creatinine ≤1.5 × age-specific upper limit of normal - Corrected serum calcium ≤11.5 mg/dl - Left ventricular ejection fraction ≥63% - SpO_2_ ≥94% (peripheral capillary oxygen saturation) - QT interval corrected by the Fridericia formula (QTcF = QT/PR^1/3^) <0.45 seconds - Life expectancy of at least 6 months from the date of enrollment - Women of childbearing potential and fertile men must agree to use highly effective contraceptive methods from the time of informed consent to at least 6 months after the last dose of lyophilized pegaspargase (for women) or from the start of lyophilized pegaspargase administration to at least 6 months after the last dose of lyophilized pegaspargase (for men). Women of childbearing potential must have a negative serum or urinary pregnancy test result at screening test   **Exclusion criteria:**   - Continuous use of corticosteroids (transient use for transfusion reactions and topical or local use for the treatment of diseases other than the primary disease will be allowed) - Positive for HBs antigen, HCV antibody, or HIV antibody. Patients who are negative for HBs antigen but positive for HBc antibody and/or HBs antibody will undergo an HBV DNA test and will be excluded from the study if they are positive for HBV DNA (≥20 IU/ml [1.3 LogIU/ml]) - Prior treatment or possible prior treatment with an L-asparaginase preparation - History of sensitivity to PEG or PEG-based drugs - Current symptoms or signs of CNS involvement (e.g., cranial nerve symptoms such as facial palsy), with CNS disease detected on CT or MRI - Pregnant (or planning to become pregnant in near future) or breastfeeding women (breastfeeding women will be excluded from the study even if they stop their breastfeeding) - History of previous malignancy, other concurrent malignancy, or secondary ALL - Other inadequacy determined by the investigator or sub-investigator |
| --- |

ALL, acute lymphoblastic leukemia; CNS, central nervous system; CT, computed tomography; HB, hepatitis B; HBV, human immunodeficiency virus antigen; HCV, hepatitis C virus; HIV, human immunodeficiency virus antigen; MRI, magnetic resonance imaging.

**Supplementary Table 3**. Backbone chemotherapy added to lyophilized pegaspargase

| - Ifosfamide (IFO) - Cyclophosphamide (CPA) - Methotrexate (MTX) - Cytarabine (Ara-C) - Mercaptopurine hydrate (6-MP) - Etoposide (VP-16) - Daunorubicin (DNR) - Pirarubicin hydrochloride (THP) - Vincristine (VCR) - Vindesine (VDS) - Dexamethasone (DEX) - Prednisolone (PSL) - Calcium folinate (LV) |
| --- |

**Supplementary Table 4.** Response rates at different stages of the study

|  | **Part 1**  **(N=3)** | **Part 2**  **(N=23)** |
| --- | --- | --- |
| **End of remission induction therapy** | n=3 | n=20* |
| ORR, n (% [95% CI]) | 3 (100.0 [29.2–100.0) | 20 (100.0 [83.2–100.0) |
| CR | 1 (33.3 [0.8–90.6]) | 6 (30.0 [11.9–54.3]) |
| CRi | 2 (66.7 [9.4–99.2]) | 14 (70.0 [45.7–88.1]) |
| **End of early consolidation therapy** | n=3 | n=21* |
| ORR, n (% [95% CI]) | 3 (100.0 [29.2–100.0) | 21 (100.0 [83.9–100.0) |
| CR | 2 (66.7 [9.4–99.2]) | 10 (47.6 [25.7–70.2]) |
| CRi | 1 (33.3 [29.2–100.0]) | 11 (52.4 [29.8–74.3]) |

*Patients were excluded due to missing evaluations at this timepoint.

CR, complete remission; CRi, complete remission with incomplete blood count recovery; ORR, overall response rate.

**Supplementary Table 5.** Plasma asparaginase activity-time concentrations by dose and by Part - remission induction (PK analysis set)

|  | **Scheduled time post EOI (h)** | | | | | | | | | |
| --- | --- | --- | --- | --- | --- | --- | --- | --- | --- | --- |
|  | Pre-dose | +0.083* | +4 | +24 | +48 | +96 | +264 | +336 | +432 | +600 |
| **Part 1 (all doses), n** | 3 | 3 | 3 | 3 | 3 | 3 | 3 | 3 | 3 | 3 |
| Mean (SD), IU/ml | 0 (0) | 1.92 (0.56) | 1.91 (0.50) | 1.65 (0.40) | 1.61 (0.51) | 1.47 (0.47) | 1.05 (0.28) | 0.90 (0.25) | 0.69 (0.20) | 0.02 (0.02) |
| **Part 2 (all doses), n** | 23 | 22 | 23 | 23 | 23 | 23 | 23 | 23 | 22 | 22 |
| Mean (SD), IU/ml | 0 (0) | 1.33 (0.30) | 1.26 (0.25) | 1.13 (0.19) | 1.08 (0.23) | 0.99 (0.18) | 0.75 (0.19) | 0.65 (0.16) | 0.50 (0.14) | 0.08 (0.03) |
| **82.5 IU/kg, n** | 4 | 4 | 4 | 4 | 4 | 4 | 4 | 4 | 4 | 4 |
| Mean (SD), IU/ml | 0 (0) | 1.24 (0.17) | 1.21 (0.11) | 1.06 (0.14) | 1.03 (0.11) | 0.98 (0.08) | 0.75 (0.13) | 0.63 (0.10) | 0.45 (0.08) | 0.09 (0.03) |
| **2500 IU/m^2^, n** | 22 | 21 | 22 | 22 | 22 | 22 | 22 | 22 | 21 | 21 |
| Mean (SD), IU/ml | 0 (0) | 1.43 (0.40) | 1.36 (0.37) | 1.22 (0.28) | 1.16 (0.33) | 1.06 (0.29) | 0.79 (0.23) | 0.68 (0.20) | 0.54 (0.16) | 0.07 (0.04) |

*5 minutes post-dose.

EOI, end of infusion; PK, pharmacokinetic; h, hours; SD, standard deviation.

**Supplementary Table 6.** Treatment-emergent adverse events related to lyophilized pegaspargase reported in ≥10% of patients

|  | **Part 1**  **(n=3)**  **n (%)** | **Part 2**  **(n=23)**  **n (%)** | **Total**  **(n=26)**  **n (%)** |
| --- | --- | --- | --- |
| Decreased blood fibrinogen | 3 (100) | 16 (69.6) | 19 (73.1) |
| Decreased antithrombin III | 3 (100) | 12 (52.2) | 15 (57.7) |
| Decreased white blood cell count | 0 (0) | 15 (65.2) | 15 (57.7) |
| Decreased platelet count | 1 (33.3) | 13 (56.5) | 14 (53.8) |
| Anemia | 0 (0) | 11 (47.8) | 11 (42.3) |
| Febrile neutropenia | 0 (0) | 11 (47.8) | 11 (42.3) |
| Vomiting | 0 (0) | 10 (43.5) | 10 (38.5) |
| Hyperproteinemia | 2 (66.7) | 8 (34.8) | 10 (38.5) |
| Alopecia | 0 (0) | 10 (43.5) | 10 (38.5) |
| Nausea | 0 (0) | 8 (34.8) | 8 (30.8) |
| Hypertriglyceridemia | 0 (0) | 8 (34.8) | 8 (30.8) |
| Decreased plasmin inhibitor | 3 (100) | 3 (13.0) | 6 (23.1) |
| Decreased plasminogen | 3 (100) | 3 (13.0) | 6 (23.1) |
| Decreased lymphocyte count | 0 (0) | 5 (21.7) | 5 (19.2) |
| Hyperlipidemia | 3 (100) | 2 (8.7) | 5 (19.2) |
| Coagulopathy | 0 (0) | 4 (17.4) | 4 (15.4) |
| Abnormal hepatic function | 0 (0) | 4 (17.4) | 4 (15.4) |
| Hypogammaglobulinemia | 0 (0) | 4 (17.4) | 4 (15.4) |
| Prolonged activated partial thromboplastin time | 1 (33.3) | 3 (13.0) | 4 (15.4) |
| Decreased neutrophil count | 0 (0) | 4 (17.4) | 4 (15.4) |
| Decreased appetite | 0 (0) | 4 (17.4) | 4 (15.4) |
| Malaise | 0 (0) | 3 (13.0) | 3 (11.5) |
| Increased alanine aminotransferase | 0 (0) | 3 (13.0) | 3 (11.5) |
| Increased aspartate aminotransferase | 0 (0) | 3 (13.0) | 3 (11.5) |
| Increased blood bilirubin | 0 (0) | 3 (13.0) | 3 (11.5) |
| Hyperglycemia | 0 (0) | 3 (13.0) | 3 (11.5) |
| Hypoalbuminemia | 0 (0) | 3 (13.0) | 3 (11.5) |

Adverse events are coded using the MedDRA dictionary, version 24.1.
